# A five year audit of ventilator-associated pneumonia: trends, organisms, antibiotic therapy and admission specialties

**DOI:** 10.1186/2197-425X-3-S1-A701

**Published:** 2015-10-01

**Authors:** F Baldwin, D O'Neill

**Affiliations:** Royal Sussex County Hospital, Brighton, United Kingdom; Intensive Care Unit, Royal Sussex County Hospital, Brighton, United Kingdom

## Introduction

Ventilator-associated pneumonia (VAP) occurs greater than 48 hours following endotracheal intubation and is a clinical diagnosis with no gold standard diagnostic criterion. The Clinical Pulmonary Infection Score (CPIS) is a tool used to aid diagnosis and comprises a clinical score of 0-12 based on 6 variables: body temperature, leukocyte count, volume and character of tracheal secretions, arterial oxygenation, chest radiograph findings and results of culture of tracheal aspirates. A score of ≥ 6 has been shown to correlate with the presence of VAP with high sensitivity although low specificity [1]

## Objectives

The aim of this audit was to use the CPIS to screen all patients ventilated in our institution over the past five years for VAP and look at trends in VAP rates over time. We also reviewed all patients diagnosed with VAP to assess causative organisms, antibiotic treatments and admission specialty.

## Methods

We used our electronic patient records to generate a CPIS comprising temperature, white blood cell count, tracheal secretions, microbiology results and PaO2/FiO2 ratio for each patient ventilator day from September 2009-August 2014. Any patient with an admission diagnosis of pneumonia was excluded. Any patient with a CPIS ≥ 4 after the first 48 hours of ventilation had their chest radiographs reviewed and scored. Patients with a score of ≥ 6 then had their electronic patient record reviewed to identify a diagnosis of either VAP or other condition.

## Results

There were 118 potential episodes of VAP identified using a CPIS ≥ 6 in patients admitted over a five year period. Review of the clinical records demonstrated 64 episodes of VAP and 54 episodes where another source of sepsis or indication for antibiotic therapy was identified. This correlates with a specificity of 54%.

**Figure 1:**
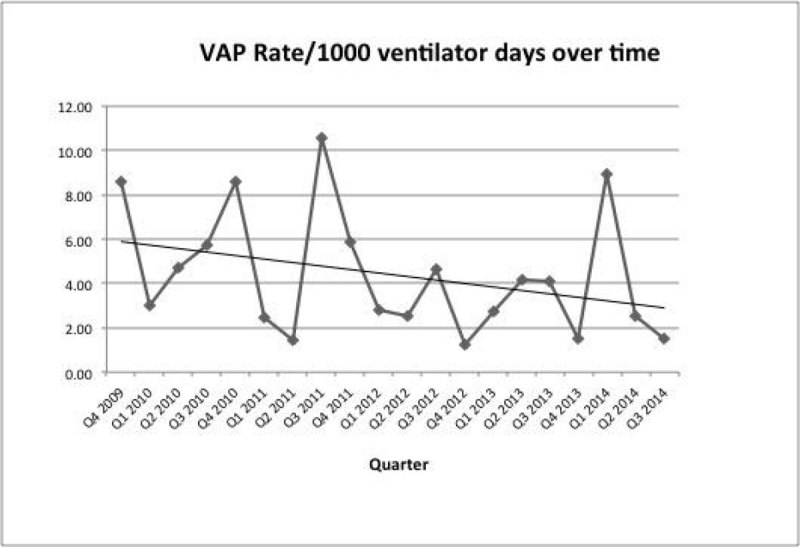
**VAP rate over time.**

Figure 1 shows a reduction in VAP rate over time.

Of all patients diagnosed with VAP there were a high percentage from certain specialties: Trauma 27%, Cardiac 18% and Vascular 17% of patients. The relative percentage of patients admitted from these specialties to our ICU in this period was 5%, 6% and 4% respectively.

**Figure 2:**
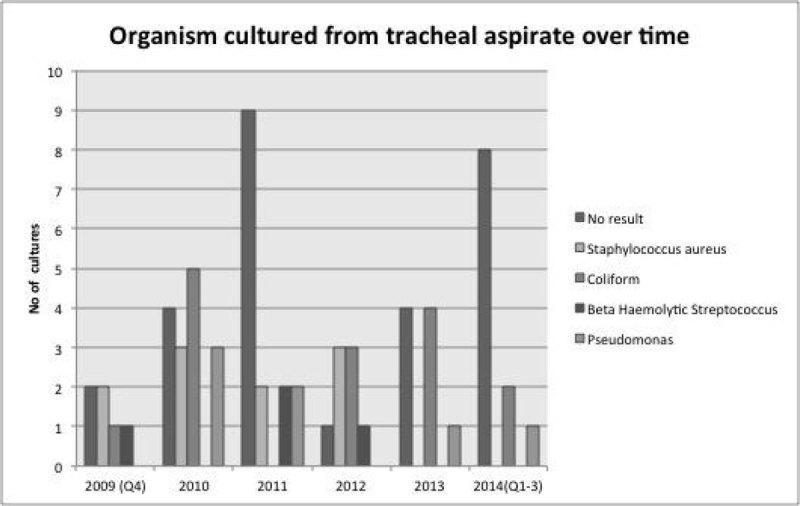
**Tracheal aspirate results over time.**

Figure 2 shows the sputum culture results over time and that peak VAP rates correlate with negative cultures.

**Figure 3:**
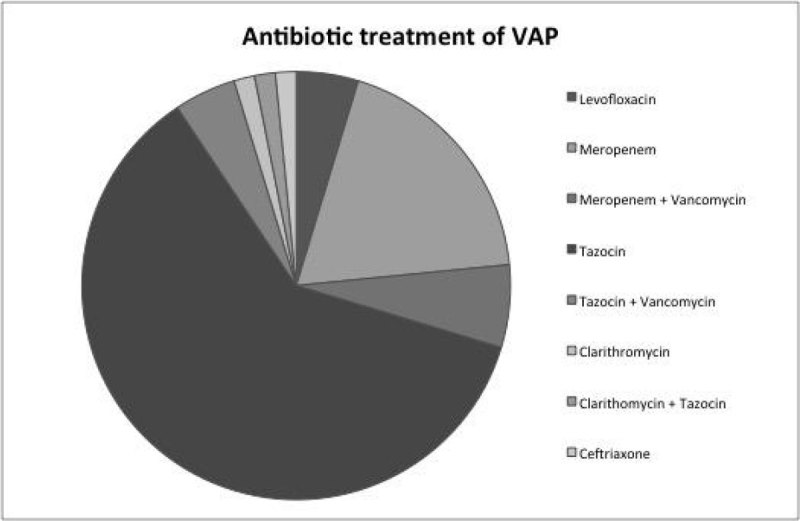
**Antibiotic treatment of VAP.**

Figure 3 shows the relative usage of antibiotics. The majority of patients received Tazocin (Piperacillin and Tazobactam).

## Conclusions

The specificity of the use of a CPIS ≥ 6 in the identification of VAP of 54 % in our audit is similar to that previously described [1]. VAP rates have reduced over time and peaks may be due to over diagnosis. The organisms identified were sensitive to the antibiotics initiated given local resistance patterns. Patient with preceding collapse were more likely to be diagnosed with VAP.
